# A Romantic Genius? The Experience of Knowledge that Shaped Werner Heisenberg's Scientific Persona

**DOI:** 10.1002/bewi.202400017

**Published:** 2025-06-16

**Authors:** Elena Schaa

**Affiliations:** ^1^ School of Religion, Theology, and Peace Studies Trinity College Dublin Dublin Ireland

**Keywords:** aesthetics of knowledge, experience of knowledge, genius, science popularization, scientific persona, Werner Heisenberg

## Abstract

In 1976, the year that Werner Heisenberg passed away, Armin Hermann published a short biography, titled *Werner Heisenberg in Selbstzeugnissen und Bilddokumenten*. Since then, historians and biographers have offered their accounts on Heisenberg's life and his contributions to modern physics. Many of these biographies present Heisenberg as a genius. Upon closer inspection, the ideal of the genius relies on the topos of the experience of knowledge presented in Heisenberg's memoir from *Der Teil und das Ganze. Gespräche im Umkreis der Atomphysik.* This article discusses the influence of this topos on his biographies. The article first contextualizes Heisenberg's popular science texts among his academic career and the cultural contexts of the German *Bildungsbürgertum.* Second, it focuses on the aesthetic repertoire of knowledge production as the experience of knowledge. By going beyond the semantic level, it is shown that the topos of the experience of nature in Heisenberg's memoir is central to his scientific persona. Ultimately, the idea of the genius stands in a *longue durée* of German Romanticism and natural philosophy is shown to shape the masculinities and scientific personae of the modern physicist.

## 1 Introduction: Telling the Story of a Heisenberg's Achievements

In 1976, the year that Werner Heisenberg passed away, Armin Hermann published a short biography, titled *Werner Heisenberg in Selbstzeugnissen und Bilddokumenten.* ^1^ A former doctoral student of Heisenberg's, Hermann had passed a draft of the biography on to Heisenberg to get his feedback. In the epilog, Hermann cites Heisenberg approval of the biography: “he wrote me, how he read many paragraphs with pleasure.”^2^ Hermann tells a story of an ambitious man who sought to gain a deeper understanding of nature by focusing on unity. Along with the insight offered into Heisenberg's research career, Hermann presents Heisenberg as a physicist who left a mark not only on the scientific community but on the political landscape of Germany.

Hermann's biography is one of three discussed in this article. These three biographies stand apart from others due to their use of the topoi presented in Heisenberg's own popular science texts. They are stories of an outstanding physicists and construct an image of Heisenberg as a Romantic genius who was at the same time an average citizen.

The second biography on Heisenberg discussed in this article was written by another of his postgraduate research students: Helmut Rechenberg. After his graduation, Rechenberg turned to the history of physics to write, among others, a scientific biography titled *Werner Heisenberg: Die Sprache der Atome: Leben und Wirken: Eine wissenschaftliche Biografie: Die* *„Fröhliche Wissenschaft“ (Jugend bis Nobelpreis*).^3^ Rechenberg concentrates on Heisenberg's professionalization and his research up until 1933, the year Heisenberg was awarded the 1932 Nobel Prize of Physics for “the formulation of quantum mechanics.”^4^

The last biography, written by the German historian‐of‐science‐turned‐science‐communicator, Ernst Peter Fischer, offers an overview of Heisenberg's whole life. Fischer explicitly aims to revitalize Heisenberg as a great scientist in this biography, called *Werner Heisenberg. Ein Wanderer zwischen zwei Welten.* ^5^ Like Hermann and Rechenberg, Fischer incorporates the topos (a common image and/or way of argumentation) of the experience of knowledge, present in Heisenberg's memoir, to construct the scientific persona of Heisenberg.

The ideal of the Romantic genius Heisenberg was complemented, however, by the idea of Heisenberg as an average German citizen. In 1983, Heisenberg's wife, Elisabeth (née Schumacher) published her recollections of her husband's life *Das politische Leben eines Unpolitischen: Erinnerungen an Werner Heisenberg*.^6^ Elisabeth's book is an attempt to restore Heisenberg's reputation by offering an explanation for his decision to stay in Germany during the Third Reich and take part in the German nuclear project, known as the *Uranverein (1939*–*45)*. This attempt to rehabilitate her husband's image comprises, in part, the presentation of him as an apolitical person, who was at the same time a genius and an “average citizen.” ^7^

He was first a spontaneous person, following that a scientific genius, next an artist close to the creative spark, and only in the last instance out of a sense of duty, a *‘homo politicus.’* ^8^

Elisabeth suggests that Heisenberg's “scientific creativity” and his occupation with the “dramatic changes of physics”, left no room for political engagements.^9^ As a result, any political aspects of Heisenberg's life appears as circumstantial rather than the result of his actions or way of being in the world. Here doing physics is presented as an apolitical practice, veiling the ideologies embedded in it.

Although Heisenberg conducted research in modern physics, which was discredited as “Jewish Physics” by fellow physicists who supported the Nazi Regime, like Johannes Stark and Philippe Lenard, Heisenberg rose through the ranks of physics in Germany to become director of the Kaiser‐Wilhelm‐Institute of Physics in Berlin and a member of the *Uranverein*.^10^ Unlike its US‐American equivalent of the Manhattan Project (1942–46), the *Uranverein* did not succeed in the development of nuclear weapons. Nevertheless, Heisenberg's involvement in the project received much media attention after the war and continues to be a subject of an ongoing debate.^11^

Following the Second World War, Heisenberg became a public advocate of science. The historian Cathryn Carson highlights the political aspects of Heisenberg's engagement and how he became, through public outreach, “Germany's public philosopher.”^12^ In the public eye as an embodied individual, Heisenberg's “scientific persona”^13^ was shaped by the arguments and the aesthetic repertoire^14^ of his popular science texts with his posture and voice bringing them to live in speeches and interviews.^15^

Biographers have furthermore highlighted the role of the culture of the German *Bildungsbürgertum* (educated bourgeoisie) in Heisenberg's biography, from his formation in Munich to the efficacy of his scientific persona after the war. In his biography on Heisenberg, the historian David Cassidy embeds Heisenberg's upbringing in the wider *bildungsbürgerliche* culture around 1900,^16^ while Carson draws attention to the activation of the *bürgerliche* ideal of the educated and integrated individual in Heisenberg's public presence in West‐Germany.^17^ Moreover, the philosophical studies on Heisenberg's popular texts illustrate the influence of the *bürgerliche* canon on arguments, but without critically assessing their ideological implications. As such, the consequences of the *Bildungsbürgertum* on Heisenberg's research and public presence often remains abstract.

The aim of this article is to show how the aesthetic repertoire of Heisenberg's texts shapes the scientific persona presented in his biographies. Central to the ideal of Heisenberg as a Romantic genius is the topos of the experience of knowledge. Shaped by Romanticism and natural philosophy, the topos aestheticizes the physicist's production of knowledge as an immediate encounter with nature, emphasizing the physicist's exception abilities.

To discuss these phenomena, I first highlight the cultural, political, and scientific contexts of Heisenberg's popular science writings. I then take a closer look at Heisenberg's memoir *Der Teil und das Ganze.* *Gespräche* *im Umkreis der Atomphysik*,^18^ in which the aforementioned topos aestheticize the particular scientific insight that introduces the reference point for matrix mechanics. I show how the topos creates an integral part of Heisenberg's scientific persona and masculinity presented in the selected biographies.

Finally, I examine the aesthetization of knowledge production and how it forges Heisenberg's scientific persona. Through the analytical concept of scientific masculinity, I highlight the cultural context within which Heisenberg was seen as a Romantic genius. Part of this *bildungsbürgerliche* culture has been the imagination of nature in the tradition of German Romanticism and natural philosophy. In Heisenberg's case the two traditions are pivotal for his public communication and his presentation of knowledge production. The masculinity of the Romantic genius, I conclude, has influenced the ideal of the theoretical physicist as an outstanding man.

## 2 A Scientific Persona in the Making: Heisenberg's Popular Science Texts

In the late 1920s, Heisenberg started what was to become his robust era of public outreach, presenting his research to the wider public through the media of speeches, articles, interviews, and books. In Heisenberg's case, this engagement in popularization of science continued a long‐standing tradition of the *Bildungsbürgertum*.

Born in Würzburg on December 5th, 1901, Werner Karl Heisenberg grew up in Germany as the second son to August and Annie (née Wecklein) Heisenberg. August was the first in his family to enter university, after which he worked as a Classics teacher and was ultimately appointed in 1910 as a professor of medieval and modern Greek philology (*Byzantinistik*) at the Ludwig Maximilian University of Munich. For the newly appointed professor August, the move south represented social advancement through education. Like August, Annie's father, Nikolaus Wecklein had entered the *Bildungsbürgertum* through both education and marriage into a family of former nobility. In Munich, Nikolaus held influential positions in the Academy of Science and the Bavarian school board. Moreover, Nikolaus was the headmaster of the humanistic Maximilian Gymnasium until 1913, which Heisenberg and his brother attended. There, the neo‐humanistic teaching focused on classics and German literature, leaving little room for mathematics, number theory, physics, or geometry.

In the German‐speaking world of the early 19th Century, educational reforms, structural changes, and cultural movements brought forward a social class that was guided by a distinct ideal of education (*Bildung*).^19^ This *Bildungsbürgertum* valued education and was entrenched with bourgeoise ideals of the citizenship. These practices and spaces were accompanied by a division of gender, with the public and workspaces becoming places where masculinities played out and the private and the household where femininities were forged and wherein the work became successively invisible. Within these dynamics, the work of the scientists and the public presentation of research became a place of masculinity. Up until the mid‐1920s, the Maximilian Gymnasium was an all‐male institution.^20^ Following his graduation from the Gymnasium, Heisenberg's professionalization continued in mostly male‐spaces. Even later on, once a recognized physicist, Heisenberg's seminars and research remained a largely all‐male environment, with some exceptions.^21^

Another of Heisenberg's formative space was among the boy scouts. Back at school after the First World War, Heisenberg became the leader of the “Heisenberg group” in 1919, a branch of the Bavarian Scout association, which bonded over short exercises (*Übungen*) around Munich and long trips (*Fahrten*) across Germany, Austria, or Finland, involving a lot of walking and some skiing. Along with physical activities, the group's diary indicates lively discussions about politics and philosophy.^22^ Furthermore, the more spiritual focus on nature was complemented with a “Christian ethic” and a nondenominational Christian theology. The ideals of the scouts, together with their physical activities and conversations, had a lasting influence on Heisenberg's worldview and persona. Prior to his wedding in 1937, Heisenberg reflected on his worldview in a letter to Wolfgang Rüdel, a friend from the scouts and his officiant, that “[m]y position towards life formed mostly during my time at the boy scouts.”^23^ The German boy scouts incorporated the Romantic perspective of nature in their practices and ideas.^24^ Which, as I will show can be found in Heisenberg's later representation of knowledge production.

Heisenberg's professional identity as a theoretical physicist was first shaped by the seminar of the theoretical physicists Arnold Sommerfeld at the Ludwig Maximilian University of Munich. At the age of 22 years old, Heisenberg finished his studies in Munich with a doctorate in theoretical physics.^25^ Afterwards, he went on to work as an assistant with Max Born in Göttingen and with Niels Bohr in Copenhagen. In 1925, he published a paper that would earn him the Nobel Prize.^26^ At only 27 years old, he held his first full professorship for theoretical physics at the University of Leipzig, had flown on an airplane,^27^ and had traveled to England, the USA, Japan, and India. Most citizens in Germany never had such experiences, clearly setting him apart from the average citizen.

With his achievements in physics, such as a paper on the uncertainty principle in 1927, Heisenberg's visibility grew beyond the eclectic circle of his colleagues in theoretical physics. The same year, Heisenberg went on to author one of his first texts^28^ addressed to a wider audience, an audience without an in‐depth knowledge or training in physics. During the 1930s, he continued to publish the occasional article and give presentations in various settings.^29^ These texts, as many that followed over the subsequent four decades, are characterized by a reflection on the practical and epistemological consequences of quantum mechanics for research and the social role of science. With the transformation of Germany in the 1930s into a totalitarian dictatorship under Adolf Hitler, the researchers who remained in Germany became more and more alienated from their colleagues around the world. During the war, Heisenberg worked for the *Uranverein*, which had significant impact on the public perception and actions following the war 1945.

At the time, Heisenberg's popular science texts revolved largely around a critique of the materialist focus of modern science, shaped by a reference to the Johann Wolfgang von Goethe's *Theory of Colour* from 1808.^30^ His interest in Goethe's work is visible in a number of speeches and posthumously published manuscripts.^31^ In the mid‐1940s the number of his speeches, interviews, and articles for a lay audience surpassed the number of academic publications.^32^ The public outreach went on to shape his later career as physicist in Germany after the war. Up until his death of cancer on February 1st, 1976, Heisenberg continued to publish popular science texts and give speeches and interviews.

Following the Second World War and the Nuclear Bombing of Nagasaki and Hiroshima, the critique of modern science changes tone to include reflections on the responsibility of the researcher and the social role of (Christian) ethics. Heisenberg signed two letters against nuclear armament in Germany, first in 1957, the *Göttinger Manifest* alongside 17 other nuclear scientists, and later in 1961, the *Tübinger Memorandum* alongside eight colleagues and friends from different areas. Heisenberg also took part in the reconstruction of the research landscape in West‐Germany. He served as the president of the Alexander von Humboldt Foundation (1953–1975) promoting international cooperation as well as the representative of West‐Germany and briefly as the vice‐President of the council of CERN (the European Organization for Nuclear Research in Geneva) (1958–1960).

Heisenberg's most prominent research of this period, is the work on unified field theory, which gained a lot of public attention at the time and is now wildly known as the *Weltformel* [Worldformula].^33^ The most notable popular writings from the time are his Gifford Lectures at St Andrews from 1954/5, which were first published in 1956 under the title *Physics and Philosophy:* *The Revolution in Modern Science.* ^34^

In 1969, Heisenberg published an even more successful book, the aformentioned memoir, *Der Teil und das Ganze*. Formatted as a collection of conversations between the protagonist, Werner Heisenberg, and his friends and colleagues, the memoir recounts different moments of Heisenberg's career and the development of modern physics set between 1919 and 1965. In the preface of the memoir, Heisenberg shares his motivations for writing the book. The book serves an invitation to the public to engage in the problems posed by modern physics.

Modern atomic physics posed anew fundamental philosophical, ethical, and political questions, and many people should engage in this discussion. Maybe the present book can also contribute to the lay of the foundation for it.^35^

The memoir won, in 1970, the Sigmund Freud Prize for Academic Prose, for the “excellent language” Heisenberg used to talk about “the difficult subject of theoretical physics” proving “the utility of language as a tool of knowledge.”^36^ The book was held in high esteem by not only the jury of the *Deutsche* *Akademie für Sprache und Dichtung* but also by general readers. Published just in time for Christmas sales, the book was heavily advertised and became a bestseller that same year.^37^ The epistemological interpretations of quantum physics and the social questions discussed along with it bring together different philosophical traditions. Biographer Carson suggests that Heisenberg's texts were successful across the board, from the *bildungsbürgerliche* to the counterculture audience. But since Heisenberg trained purely in theoretical physics, his popular science texts remain “amateur philosophizing.”^38^ It is his achievements in theoretical physics that grant him the authority to speak on subjects that lay beyond his professional expertise.

Heisenberg's popular science texts include explicit reflections on science in dialog with topics, such as religion, epistemological questions, or social critique. Starting in the mid‐1930s, the critique of materialist science is entwined with a welcome reception of natural philosophy, especially von Goethe's work. Over the decades, some of these positions undergo fundamental changes, for example, the call for Christian ethics as the framework to evaluate research goals,^39^ while other views remain stable, for example, the hierarchy between understanding and predicting.^40^ Moreover, the aesthetic repertoire of these texts are integral for his interpretation of knowledge production. As I will show, in the memoir, Heisenberg's breakthrough in quantum mechanics uses the topos of the experience of nature, resulting in the presentation of knowledge production as an experience of knowledge.

## 3 A Stroke of a Genius: Experiencing the Mathematics of the Matrix Mechanics

One such topos is the development of the link between experience of nature and the production of knowledge. In the chapter “Quantum Mechanics and a Talk with Einstein (1925–1926)” Heisenberg recounts the breakthrough that lead to the formulation of matrix mechanics. The chapter opens with a description of the state of “quantum mechanics”^41^ in the early 1920s and links it to a hike with his scout friends in the Austrian Alps in 1924: “When I think about the state of atomic theory in those months, I always remember a mountain walk.”^42^

His description of the scouts climbing Guffert mountain is picturesque as they walk through “an obscure tangle of rocks and slopes” and lose sight of one another in “the moving wafts of mist” to find their way again once they “see the edge of a steep rock, straight ahead and bathed in bright sunlight.” Persisting with their climb, the scouts are rewarded with a view over the Alps from “the sunny saddle heights above the sea of fog.”^43^ Here mountaineering presents a mode through which humans encounter nature. As a cultural practice, the encounter with nature through mountaineering can be one of domination or of revelation.^44^ Romantic authors, especially, describe the encounters with a mountain as an epiphany of sacred dimension of nature, its living beauty condensed in the *natura naturans (nature naturing).* ^45^ In many cases, the revelation of the sacred dimension of nature is accompanied with the description of looking over the land from above, which offers the observer a new perspective on the landscape. This topos was put on canvas by Caspar David Friedrich, and since then, the painting “Wanderer over the sea of fog” (c. 1818) has become paradigmatic. The fog, partly veiling the land in Friedrich's painting, heightens the role of the imagination and renders the scene insubstantial by eradicating any connection with the ground. The central figure, with his back to the observer, reminds us of the sublime and a longing for unity with the infinite.

This scene of Heisenberg's experience of nature is followed by an interlude about the protagonist's research, which he describes as an “impenetrable thicket of complicated mathematical formulas.”^46^ Furthermore, the interlude links the hike and the state of physics with the protagonist's breakthrough in modern physics during a trip to the island Helgoland. The chapter ends with a discussion between the protagonist and the character Einstein on the epistemological consequences and empirical validity of the newly presented mechanics.

The central moment of knowledge production in the memoir is set on the remote island of Helgoland off the coast of Germany, which Heisenberg sought out to recover from his hay fever.^47^ It is the work he conducts in Helgoland that leads to the seminal papers on the matrix mechanics that are foundational to quantum mechanics. Here, the topos of the experience of nature is entwined with the production of knowledge to become the experience of knowledge.

At the end of May 1925, I fell so ill with hay fever that I had to ask Born to release me from my duties for 14 days. I wanted to travel to Helgoland, to cure my hay fever with sea air, far away from blooming bushes and meadows. When I sat on my balcony, I had many opportunities to think of Bohr's comment that the view over the sea allows us to grasp a part of infinity.

Apart from daily walks and long swims, there was nothing in Helgoland to distract me from my problem, and so I made much swifter progress than I would have made in Göttingen. A few days were enough to jettison all the mathematical baggage that accumulates at the beginning and to arrive at a simple mathematical formulation of my question. […] I concentrated on the question of the conservation law's validity, and one evening I reached the point where I was ready to determine the individual terms in the energy table, or, as we put it today, in the energy matrix, by what would now be considered an extremely clumsy series of calculations. Once the first term really confirmed the energy principle, I got so agitated that I made countless arithmetical errors. As a result, it was almost three o'clock in the morning before the final result of my calculations lay before me. All terms validated the energy principle and since the results presented themselves, so to say without any effort, I could no longer doubt the mathematical consistency and coherence of the indicated quantum mechanics. At first, I was deeply startled [*zutiefst erschrocken*]. I had the feeling that through the surface of atomic phenomena, I was looking at an underlying ground [*darunter liegenden Grund*] of strange inner beauty. I felt almost giddy at the thought of the wealth of mathematical structures, I now had to pursue. I was far too excited to sleep. So, I left the house at dawn and headed to the southern tip of the Oberland, where a lone rock jutting out into the sea had always whetted my desire for climbing. I managed to climb the tower without too much trouble and awaited the sunrise on its top.

What I saw during that night in Helgoland was admittedly not very much more than the sunlit rock edge I had glimpsed at in the autumn of 1924.^48^

The passage of the breakthrough in Helgoland stretches over two pages, and during which, we find different narrative registers. Starting with the descriptions of the circumstances and Heisenberg's mode of working, the protagonist goes on to describe the mathematical problem he is working on. The attempt to solve the problem turns into a figure of sensation culminating in the momentary experience of knowledge, in particular mathematical structures.

The change of register to one of sensation stands in the tradition of the “modern aesthetic religion”^49^ for which Friedrich Schleiermacher paved the way with his redefinition of religion as “intuition and feeling” and an “intuition of the universe.”^50^ The somatic changes and affective language of this passage present the moment of knowledge production as one of “overwhelming sensations.”^51^ The topos of the experience of nature turns here into the experience of knowledge, which serves here as confirmation for the validity of his work. In the conversation with Einstein, the protagonist describes the experience in the following way: “Nature leads us to mathematical forms of great simplicity and beauty.”^52^ Here, the combination of sensations, such as effortlessness and immediacy as well as aesthetic judgments of beauty and location of the theories present nonepistemic values of knowledge production.^53^ The experience develops a correspondence between the hidden forces of nature, with the beautiful ground and mathematical formula on one side, and on the other, the imaginative genius within the human that emerges in the absence of worldly distractions. Similar to the experience of nature in the mountains, the encounter with nature is framed by the longing for unity with the infinite. Induced by the sight from the balcony and the awaiting of the sunrise, the longing for that unity is an individual experience.

The individuality of this moment of knowledge production stands in contrast to the premise of the memoir that “science is created in conversations.”^54^ This idea of science as a collaborative practice is emphasized by the memoir's structure as a series of conversations. This idea, however, does not apply to Heisenberg's breakthrough in the quantum mechanics. While the experience of knowledge stretches over two pages, the collaboration between Heisenberg, Max Born, and Pascal Jordan that leads to the publication of the three seminal papers is explicitly excluded: “Of the extremely intensive work which kept us breathless for a few months I won't report.”^55^ The focus of the individual experience of knowledge as the key moment of knowledge production is crucial for the ideal of the Romantic genius. The German Physics Society and the Max Plank Institute for Theoretical Physics erected in June 2000 a rock on Helgoland to commemorate Heisenberg's breakthrough in modern physics.^56^

The three biographies incorporate the experience of knowledge in different ways in the presentation of Heisenberg's persona. Fischer models his biography around the topos of the experience of knowledge through mountaineering. The topos appear already in the title *Wander zwischen den Welten* ^57^ and shapes the first chapter, “*Einblick: der Wanderer*.”^58^ In the chapter and throughout the book, Fischer characterizes Heisenberg as a person who had the ability to *wandern* [wander or hike], from hiking in the mountains to wandering in physics, while connecting these worlds by wandering between them.^59^ Fischer identifies this ability to wander between different social systems with Heisenberg's public engagement, in which he “described atoms and quantum leaps in a plain language.”^60^ The wandering foreshadows the imaginative genius within Heisenberg: “One night, his whirling thoughts bring him very close to the atoms and their secret, suddenly a new dimension of the world of numbers opens up inside him.”^61^ While the experience of knowledge in Heisenberg's memoir is located outside of him, Fischer heightens the individuality of this experience, and with it Heisenberg's imaginative genius, by locating the secrets inside of him.

The experience of knowledge takes center stage in Fischer's book as the second chapter, “*Eine Nacht auf Helgoland*.”^62^ The night in Helgoland is presented as a turning point for Heisenberg: “he lived no longer as a sad guest [*trüber Gast*] on the dark earth […] Heisenberg had become the companion [*Wegbegleiter*] of a new view of reality.”^63^ While in Heisenberg's memoir, the experience of knowledge is tied to modern physics, in Fischer's biography, it becomes a turning point in Heisenberg's life, similar to an epiphany. In this biography, the topos is a central piece of the “forgotten genius,”^64^ and Heisenberg is made out to be “one of the most important thinkers of the 20th Century.”^65^

In Hermann's biography, the topos of the experience of knowledge is combined with the metaphors of the *terra incognita* and the building of bridges: “Heisenberg built a bridge and stepped on the new land.” Hermann further draws on the metaphor of piety by setting the breakthrough in a “seclusion” that allowed Heisenberg to focus on “the essence” of the mathematical problem. Rather than referring to Heisenberg's memoir, Hermann interprets the breakthrough by citing from an undated conversation between Heisenberg and Bartel Leendert van der Waerden in which Heisenberg describes the experience as an epiphany: “There was a moment in Helgoland, in which it came to me like an epiphany [*Erleuchtung*].”^66^ While Fischer uses the narrative of an epiphany as a turning point, Hermann embeds the epiphany in the wider context of contemporary research in physics. The research that brought forth the seminal papers on matrix mechanics is characterized as “the genius’ obsession with its object.”^67^ Unlike Fischer, Hermann emphasizes Heisenberg's “nice and humble” character with a continuously balanced “sense of facts and intuition.”^68^ As a result, Heisenberg's scientific persona is one of a public speaker with the ability to capture his audience's attention with “unusual intellectual clarity” and “serenity.”^69^ Hermann's biography draws the attention to the public efficacy of Heisenberg's persona and builds on the communal and political aspects of his life.

Rechenberg's biography, by contrast, focuses on Heisenberg's academic work. Embedded in the discussion of the esoteric knowledge, Heisenberg worked on in 1925, Rechenberg cites the passage from Heisenberg's memoir as evidence for the “dramatic events” brought by his stay in Helgoland.^70^ Furthermore, the relevant chapter is titled “*Der Durchbruch zur Quantenmechanik in Helgoland*”^71^ and includes a formulation that resembles the above passage from Heisenberg's memoir. We find the shift from agony to ease once Heisenberg reaches Helgoland, “in the pollen‐free air of Helgoland, he immediately recognized how he had to use Sommerfeld's integral,”^72^ or “naturally this uncertainty [*Unbestimmtheit*] annoyed Heisenberg in June 1925, but on Helgoland he easily found the answer.”^73^ The topos is present in Rechenberg's biography, although it is less prominent than in Fischer's biography and even more embedded in the discussion of Heisenberg's research then Hermann's book. Together, they contribute to Rechenberg's construction of Heisenberg scientific persona as a Romantic genius, which is most poignant in his description of Heisenberg as “a juvenile genius”^74^ who “finalized quantum theory” during the “the golden age” of physics.^75^ The three biographies use the topos of the experience of knowledge to tell their stories of Heisenberg's life and in particular his academic achievements.

## 4 Heisenberg's Scientific Persona: The Romantic Genius and Critical Scientist

In Heisenberg's memoir, it is the experience of knowledge that stands at the core of the formulation of the newly established matrix mechanics. As a topos, this experience of knowledge goes on to shape Heisenberg's scientific persona as represented in the three biographies. While the biography of Fischer is fully modeled around the experience, Hermann's and Rechenberg's books embed, albeit differently, the topos in the history of Heisenberg's research, wherein the encounters with nature contribute to the construction of Heisenberg as a genius.

The ideal of the genius stands in the *longue durée* of the cultural movement of Romanticism and has thus also been referred to as the “Romantic genius.”^76^ At the time, central qualities of the genius were a somatic and mental sensitivity toward nature which made the encounters with nature possible in the first place. Along with the individual's predisposition, poetry was said to facilitate immediate understanding and offer a form to capture and imagine this cognitive and emotive connections.^77^ In taking a closer look at the ideal through the analytical framework of “scientific masculinity,”^78^ we can see that the aesthetics of the genius creates a relationship between male research and female nature. The (Romantic) genius has challenged and has been challenged by other masculinities, such as the engineer,^79^ the scholar and the gentlemen of science,^80^ or the professional scientist.^81^ In Heisenberg's case, the scientific persona is entwined with a masculinity that builds on sensitivity toward nature, much like the genius of the Romantics. This masculinity was recognized by the *Bildungsbürgertum* and presented even an alternative to the “inhuman scientist,” who was guided by materialist ideals^82^ or aristocratic masculinities.^83^

The “scientific persona” captures the “intermediate between the individual biographies and the social institution.”^84^ As such, it presents a cultural identity, which for scientists is based on the profession or the public intellectual, a public role. This cultural identity shapes the individual both on the somatic and psychological levels, while creating a collective with a shared and recognizable physiognomy. Personae are creations of historical circumstance and material conditions, and as such emerge and disappear; they tell us a story about social dynamics, collective ways of thinking, working, feeling, interests, and meaning‐making. In Heisenberg's case, the scientific persona is the result of the combination of multiple roles masculinities and even roles, such the theoretical physicist, the public speaker, or nature lover.

Through the lens of masculinity studies, we can see that the ideal of the genius is by no means ideologically indifferent, and neither is the theoretical physicist engaging in public outreach an apolitical persona. However, this example shows that the confirmation of Heisenberg's texts and actions with the culture of the *Bildungsbürgertum* renders the political side of his life and way of doing physics invisible. The public discussion of in topics, such as music beyond his expertise in theoretical physics, is presented as a confirmation of his greatness, most prominently by Fischer's idea of Heisenberg as a wanderer between worlds. The role of the public intellectual, who contributes to the holistic education of the *Bürger*, resonates with the German concept of *Bildung* and citizenship.

Through his public communication, Heisenberg integrated several roles—that of the physicist, the philosophical thinking man, the nature lover, or the citizen—into one coherent persona. Carson suggests that the commitment to the ideal of a coherent individual was tied to the attempt in his texts “to put science and context back together.” The aspiration to integrate the differentiated systems of modern society and the disciplines of modern science, presents a critique of modernity. The development of *bildungsbürgerliche* culture was accompanied with a critique of modernity, enlightenment, and rationalization.^85^ The experience of nature presents the “the sensibilities of the individual” as an alternative to the observed prevalence of materialism.^86^

With the presentation of an alternative to a materialistic science and the accompanying masculinities, Heisenberg's critique places him in the *longue durée* of the critical scientist. One reference point of the critical scientist is Johann Wolfgang von Goethe, who Heisenberg explicitly references in his texts. The natural philosopher, like Goethe, praised the immediate access to nature and his ability to connect it with “other realms of his experience” as the superior practice of knowledge production.^87^ Furthermore, the critical scientists paired the “literary intellectual” with the “liberal *bildungsbürgerliche* practitioner.”^88^ Heisenberg's philosophical commentaries and public engagement promoted a *bildungsbürgerliche* ideal of science, offering answers to epistemological questions and serving political deliberation.^89^ Carson suggests that the effectiveness of Heisenberg's critical stance was the wide resonance in postwar Germany of “the critical scientist” and the 19th Century ideals at large.^90^ A number of physicists involved in the formulation of quantum mechanics presented their interpretation of the events and their roles therein in highly diverse publications.^91^

I showed how the ideal the (Romantic) genius is embedded in social and cultural contexts, using the analytical category of scientific masculinity and the concept of the scientific persona, together with the focus on the aesthetic repertoire employed by the stories of Heisenberg's knowledge production, they highlight the historicity of the genius. In Heisenberg's case, the scientific persona built on the genius and coherent scientist is the product of powerful aesthetic repertories and a bildungsbürgerliche culture that continues to shape ideals of today's physicists, even those beyond the German‐speaking world.

## 5 Conclusion: A Romantic Genius for Theoretical Physics?

Year 2025 marks the centenary of the formulation of matrix mechanics. While the three papers at the core of matrix mechanics, and with them, the foundation of quantum mechanics involving the work of three physicists—Max Born, Paul Dirac, and Werner Heisenberg—it was Heisenberg who won, eight years later, the Nobel Prize of 1932 for “the formulation of quantum mechanics.” However, in Heisenberg's memoir, a highly individual experience of knowledge stands at the beginning of the formulation of matrix mechanics. I showed, in this article, how the topos of the experience of knowledge becomes a central point for the construction of the scientific persona across three biographies.

The presentation of Heisenberg as a genius rests on the overwhelming sensation at the encounter of nature. His decision to remain in Germany throughout the Third Reich and even work for the *Uranverein* stained Heisenberg's image as a stellar physicist after the Second World War. However, his engagement in the public sphere of West‐Germany, through speeches, publications, or interviews, has shaped the scientific persona of Heisenberg as a model for citizenship. The combination of the Romantic genius with the *bildungsbürgerliche* practices of science communication placed him right at the heart of *bildungsbürgerliche* culture. For the counterculture around the 1970s, in particular in the USA, Heisenberg's persona presented a welcome alternative to the professional scientist.^92^

Even today, the experience of knowledge shapes the ideal of the physicist^93^ with far reaching consequences for physics as a discipline.^94^ This article showed how the topos of the experience of knowledge is created and transported by the popular science texts of physicists and reiterated by their biographers who tell the stories of a genius and model citizen. Ultimately, the article points to the influence that scientists and historians of science have in shaping the narratives of knowledge production and the representation of scientists.

## Conflict of Interest

The authors declare no conflict of interest.

## Data Availability

Research data are not shared.

## Footnotes

1 Hermann 1976, English translation: Werner Heisenberg. Testimonies and Pictures, E.S.

2 Hermann 1976, on p. 148.

3 Rechenberg 2010, English translation: Werner Heisenberg: The Language of Atoms, Life and Work, A scientific biography, The “joyful science” (Youth until Nobel Prize), E.S.

4 The Nobel Prize in Physics 1932. *NobelPrize.Org* 1932.

5 Fischer 2015, English translation: Werner Heisenberg: A Wanderer between two Worlds, E.S. Fischer makes his ambition explicit in the first sentences of the preface: “I have a dream. I dream that in Germany one talks more about Werner Heisenberg and less about Martin Heidegger, who both lived until 1976” Fischer 2015, on p. v, E.S.

6 Heisenberg 1983, English Translation: The political life of an unpolitical man. Recollections of Werner Heisenberg. E.S., The reference to politics is not reflected in the titled of the English translation from Heisenberg 1984, *Inner Exile. Recollections of a Life with Werner Heisenberg,* which rather alludes to Heisenberg's academic alienation beyond the Third Reich.

7 Heisenberg 1983.

8 Heisenberg 1983, here the English translation from 1984, by S. Capperalli and C. Morries. The idea of Heisenberg incorporating different roles can be found most prominently in Geyer et al. 1993.

9 Heisenberg 1983, on pp. 40, 47.

10 Cassidy 1992, on p. 234.

11 Published first in 1956: Jungk 2020, see further Rose 1998, Powers 2000, Hoffmann 2023.

12 Carson 2010, on p. 7.

13 Daston and Sibum 2003.

14 In the tradition of the aesthetics of knowledge as presented by Borrelli and Grieser 2017 and the aesthetics of religion by Grieser and Johnston 2017. The aesthetic repertoire may include among the aesthetic judgments (e.g., beautiful formulae), sensory qualities of experience (e.g., feeling dizzy or agitated), narrative structures, practices or images.

15 Carson 2003; Carson 2010, on p. 261.

16 Cassidy 1992.

17 Carson 2010.

18 Heisenberg 1985, English translation: “The many and the one. Conversations in the Environment of Particle Physics,” trans, E.S. The memoir's English translation, Heisenberg 1971, by Jan Pomerans was published in 1971 by Harper and Row and remains today the standard translation. However, much of the aesthetic repertoire that contributes to the scientific persona of Heisenberg, which is discussed in this article, did not make it into the English translation. Most of the translations used for this article are mine, and wherever possible, existing translations have been consulted, as acknowledged in the bibliography.

19
*Bildung* involved a knowledge‐ and practice‐based ‘active self‐formation’ and ‘self‐realization’ inspired by neo‐humanist ideals and which was guided by the study of texts, attendance of lectures, music‐making, or physical activities, see Klein 2020.

20 This changed when the first female teachers were employed, and female students were admitted later in the 1920s, Thomas Bednar private communication with author, 30 July 2020.

21 Some pictures printed in Hermann's biography show females, however the University Archive of Leipzig was not able to produce any records of female students, however the Rechenberg and Wiemers 2002 document in that Heisenberg gave a report for the doctorate of Elisabeth Geest, on pp. 74–75. The University Archive of Leipzig holds no records for Grete Hermann visit of Heisenberg's group. The visit covers one chapter of the Memoir, however the absent of record suggest that she visited Heisenberg's seminar in an informal manner. Annette Vogt 2008 documents the female students and researchers who worked at the Kaiser Wilhelm Gesellschaft. The physicists Brigitte Gysae worked with Heisenberg on the nuclear research project, Vogt 2008, on pp. 66. Birgit Kolboske 2023 documents in her book from 2023 the career of the physicists and mathematician Eleonore Trefftz a the Max Planck Insitute (MPI) for Physics and Astrophysics. The employment records of the MPI for Physics and Astrophysics document the progress of one female laboratory assistant to research student, Lucie Hackenbroich, nee Brusian. The student never took her doctoral exam (University Archive München, UAM, Munich Stud‐Kart III Hackenbroich, Lucie), however, the records show that the student went on to marry a colleague (Archiv der Max Planck Gesellschaft, Berlin II. Abt., Rep. 67, Nr. 175 und III. Abt., Rep. 93).

22 Becker 2019.

23 Heisenberg to Rüdel 6.4.1937 in Becker 2019, on pp. 449–450.

24 Kühne 1998.

25 The professors present at the doctoral exam were Arnold Sommerfeld and Wilhelm Wien for physics and Oskar Perron for mathematics and Hugo von Seeliger for astronomy, University Archive München (UAM), O‐I‐49p (Heisenberg, Werner).

26 Heisenberg 1925, along with Born and Jordan 1925 and Born et al. 1926.

27 Heisenberg to his parents 15.09.1926, in Hirsch‐Heisenberg 2003, on p. 110.

28 Heisenberg 1984h.

29 Heisenberg 1984f, Heisenberg 1984i.

30 Goethe 2002.

31 Heisenberg 1984a, Heisenberg 1984b, Heisenberg 1984c, Heisenberg 1984d.

32 Cassidy and Baker 1984.

33 Carson 2010.

34 Heisenberg 2000, A German translation was published by the publishing house S. Hirzel in 1959, *titled Physik und Philosophie*, Heisenberg 1984j.

35 Heisenberg 1985, on p. 9.

36 Storz et al. 1970.

37 Carson 2010, on pp. 144–150.

38 Asprem 2015.

39 Schaa 2004, on pp. 103–110.

40 Schaa 2004, on pp. 178–187.

41 Heisenberg 1985, on p. 86.

42 Heisenberg 1985, on p. 86. The notes of a fellow scout, Eberhard Rüdel, confirm the hike, including the rainy weather and the sunshine at the top and has been reprinted in Becker 2019, on pp. 339–340.

43 Heisenberg 1985, on p. 86.

44 Von Stuckrad 2005.

45 Steigerwald 2005.

46 Heisenberg 1985, on p. 87.

47 Heisenberg's escape to Helgoland to recover from a hay fever attack in June of 1925 is documented in letters to his mother. The letters further document severe hay fever attacks over the year and the different treatments Heisenberg sought. Heisenberg writes to his mother on June 5th, 1924. “I have neutralized my hay fever with aspirin” or a day later June 6th, 1924 “in this dreadfully beautiful weather, I'm eking out my miserable existence on aspirin, cocaine and similar stuff” both are edited in Hirsch‐Heisenberg 2003, on pp. 74–75.

48 Heisenberg 1985, on pp. 89–91, trans. E.S.

49 Grieser 2017, on p. 258.

50 Schleiermacher 1996, on p. 24.

51 Grieser 2017, on p. 258, for this overwhelming sensation see Schleiermacher 1996, on pp. 29–32.

52 Heisenberg 1985, on pp. 98–99.

53 Rooney 2017. The aesthetization of the mathematical formulae gives a form to the structural realism observed in Heisenberg's texts Camilieri 2009.

54 Heisenberg 1985, on p. 9.

55 Heisenberg 1985, on p. 90.

56 The Inscription states “In June 1925, here on Helgoland, the 23‐year‐old Werner Heisenberg made the breakthrough in the formulation of quantum mechanics, the fundamental theory of the laws of nature [*Grundlegenden Theorie der Naturgesetze*] in the atomic realm that has profoundly [*tiefgehend*] influenced human thinking far beyond physics”.

57 The title of Fischers's biography shows a great resemblance to the autobiographical work by Walter Flex 1916 titled *Der Wanderer zwischen beiden Welten: Ein Kriegserlebnis* [The Wanderer between both Worlds: A Wartime Experience] from 1916. From the letters to his parents, we can deduce that Heisenberg knew of the book and most likely read it, as he asks his parents in a letter from November 19th, 1924, to gift him the book for his 24th Birthday, with the note, “I need some ‘immature idealism’” in Hirsch‐Heisenberg 2003, on p. 82.

58 English translation: “Insight. The wanderer,” E.S.

59 Fischer 2015, on p. 2.

60 Fischer 2015, on p. 1.

61 Fischer 2015, on p. 3.

62 English translation: “One Night in Helgoland” E.S.

63 Fischer 2015, on pp. 14–15.

64 Fischer 2015, on vf.

65 Fischer 2015, on back cover.

66 Hermann 1976, on p. 31, E.S. The quotation seems to be from the 1960s and can be found in van der Waerden 1967, on p. 25.

67 Hermann 1976, on p. 32.

68 Hermann 1976, on p. 115.

69 Hermann 1976, on pp. 109–110.

70 Rechenberg 2010, on p. 333.

71 English translation: “The breakthrough to Quantum Mechanics on Helgoland” E.S.

72 Rechenberg 2010, on p. 325.

73 Rechenberg 2010, on p. 327.

74 Rechenberg 2010, on p. 950.

75 Rechenberg 2010, on p. vi.

76 Borrelli 2020.

77 Schmidt 1985, von Stuckrad 2009, on p. 452.

78 Milam and Nye 2015 coined the concept of scientific masculinity in the tradition of the article “Gender: A Useful Categort of Historical Analysis” from 1986 by Joan Wallace Scott 1986. Together with Milam and Nye's proposal, the focus on masculinity and science highlighted the potential of the category for the study of institutional structures, research practices, vocabulary, or ideals as sides of production, manifestation, and negotiation of masculinity and knowledge, for example, Golinski 1999 and Schnicke 2017.

79 Paulitz 2012.

80 Ellis 2017; Shapin 1991; Shapin 2006.

81 Lucier 2009. Furthermore, Köhne 2020 illustrates with a collection the plurality figured of genius in knowledge creation and Köhne und Ventarola 2022 showed how the masculinity of the genius has been contested by women both researchers and historical actors.

82 Carson 2010, on p. 143.

83 Mosse 1996.

84 Daston and Sibum 2003, on p. 2.

85 Frie 2001.

86 Carson 2003, on p. 261.

87 Carson 2003, on p. 244.

88 Carson 2003, on p. 256.

89 Carson 2010, on p. 33.

90 Carson 2003; Daston and Sibum 2003.

91 Gladigow 1986.

92 Kaiser 2012.

93 Most prominently, Rovelli 2021.

94 Leslie et al. 2015.
